# Leveraging interpretable machine learning to identify sarcopenia in middle-aged and older adults with intrinsic capacity decline: an analysis of CHARLS data under AWGS 2025

**DOI:** 10.1186/s12911-026-03606-x

**Published:** 2026-06-03

**Authors:** Jiatong Li, Runjia Liu, Enshuo Shi, Zhuorui Han, Juncheng Luo, Mingtao Wen, Jinbao Liu, Huapeng Guan, Nianhu Li

**Affiliations:** 1 First Clinical Medical College, Shandong University of Traditional Chinese Medicine, Jinan, Shandong 250014 China; 2 Department of Cardiology, Guang’anmen Hospital, China Academy of Chinese Medical Sciences, Beijing, 100053 China; 3 Affiliated Hospital of Shandong University of Traditional Chinese Medicine, Jinan, Shandong 250014 China

**Keywords:** Sarcopenia, Intrinsic capacity, Machine learning, China Health and Retirement Longitudinal Study, SHAP analysis

## Abstract

**Background:**

This study leverages machine learning to develop and validate an interpretable diagnostic model for sarcopenia, specifically tailored to community-dwelling middle-aged and older adults exhibiting intrinsic capacity decline.

**Methods:**

This study used cross-sectional data from the 2015 China Health and Retirement Longitudinal Study (CHARLS), which included 6,134 participants aged 50 or above. We employed seven machine learning algorithms to construct classification models for sarcopenia status in individuals exhibiting declined intrinsic capacity. The models’ performance was comprehensively evaluated based on their discriminative ability, calibration, and clinical applicability. Specifically, we used the area under the receiver operating characteristic curve (AUC), calibration curves, and clinical decision curve analysis for this evaluation.

**Results:**

Among the 6,134 included middle-aged and older adults with declined intrinsic capacity, 663 participants (10.81%) were diagnosed with sarcopenia according to the AWGS 2025 criteria. We performed a two-step feature selection procedure: first, 12 candidate predictors were identified via LASSO regression; subsequently, 8 core predictors significantly associated with sarcopenia risk were finally determined using multivariate logistic regression, including sex, residence, near vision ability, social isolation, activities of daily living (ADL), lower body mobility, time for walking speed test, and predictive skeletal muscle mass index (pSMI). Using these 8 core predictors as input features, we constructed and validated seven machine learning models. The XGBoost model achieved the optimal performance on the independent test set, with an AUC of 0.807 (95% CI: 0.777–0.836), a sensitivity of 55.1%, a specificity of 85.6%, and an accuracy of 82.3%. Furthermore, we applied SHAP (SHapley Additive exPlanations) analysis to decipher the black box of the model, clarify the contribution direction and magnitude of each predictor, and ensure the good interpretability of the optimal model.

**Conclusion:**

This study developed and validated a machine learning model to provide a scientific basis for health management strategy formulation. The model aims to enable early identification of sarcopenia status in middle-aged and older adults with declined intrinsic capacity. This could enhance their quality of life and alleviate associated social and familial care burdens.

**Clinical trial number:**

Not applicable.

**Supplementary Information:**

The online version contains supplementary material available at 10.1186/s12911-026-03606-x.

## Introduction

Sarcopenia, a progressive, age-related skeletal muscle disorder, has emerged as a critical public health challenge amid global population aging [1]. Worldwide, its prevalence among individuals aged 60 years and older ranges from 10% to 27% [2]. This condition is strongly associated with adverse outcomes, including an increased risk of falls, functional disability, hospitalization, and mortality [3, 4]. Recognizing the urgent need for early identification and intervention, the Asian Working Group for Sarcopenia (AWGS) 2025 Consensus Update has introduced a paradigm shift in the definition and management of sarcopenia. This update represents a pivotal advancement in the field [5].

The AWGS 2025 Consensus redefines sarcopenia as a disease characterized by the concurrent presence of low muscle mass and low muscle strength. This revision simplifies the diagnostic criteria by removing physical performance as a mandatory component; it is now classified as an outcome indicator. Critically, this adjustment to the diagnostic workflow does not diminish the pathophysiological or clinical value of physical performance metrics in sarcopenia prevention: while they are no longer required for definitive diagnosis, declines in walking speed, lower body mobility, and other physical performance parameters are well-documented early, preclinical manifestations of skeletal muscle deterioration. These changes often occur before the loss of muscle mass and strength reaches the diagnostic threshold for sarcopenia, making them ideal candidate markers for early risk prediction. This updated definition also extends coverage to middle-aged adults (aged 50–64 years) using population-specific cutoff values, thereby emphasizing early detection and intervention across the life course. A key highlight of the AWGS 2025 Consensus is its explicit emphasis on the close relationship between intrinsic capacity (IC) and sarcopenia. Defined by the World Health Organization (WHO) as the composite of an individual’s physical and mental capacities across cognitive, locomotor, vitality, sensory, and psychological domains, IC forms the foundation for healthy aging and functional independence [6]. Physical performance metrics are the direct, objective quantitative measures of the locomotor domain of IC. For our target population of adults with declined IC, these metrics not only reflect the core impairment of the locomotor domain, but also serve as early sentinels of the bidirectional vicious cycle between IC decline and sarcopenia progression highlighted in the AWGS 2025 Consensus. The AWGS 2025 Consensus posits that declined IC is not only a key risk factor for sarcopenia but also engages in a bidirectional relationship with muscle health. Specifically, declined IC compromises the maintenance of muscle mass and strength, whereas sarcopenia, in turn, exacerbates IC deterioration. This interaction forms a vicious cycle that accelerates functional decline [7]. Consequently, IC is positioned as a central target for sarcopenia risk stratification and prevention, aligning with the WHO’s Integrated Care for Older People (ICOPE) framework [8, 9].

Despite the advancements outlined in the AWGS 2025 Consensus, critical gaps remain in translating these insights into clinically practical risk stratification tools. Traditional statistical methods (e.g., conventional logistic regression) currently used for sarcopenia risk prediction are limited in their ability to capture the complex, nonlinear relationships among IC domains, demographic factors, and sarcopenia development [10–12]. However, machine learning (ML) algorithms, which excel at handling multidimensional data and identifying latent feature interactions, have demonstrated great potential in medical prediction. Despite this, ML remains underutilized in sarcopenia research, particularly for models based on the latest AWGS 2025 criteria. Second, existing prediction models lack specificity for populations with declined IC, a high-risk subgroup explicitly prioritized in the AWGS 2025 Consensus. Most available models are developed for general older adult populations, with IC status only included as an auxiliary covariate, rather than targeting this high-risk subgroup specifically. The pathophysiological mechanisms and risk profiles of sarcopenia differ fundamentally between individuals with compromised IC and those with preserved IC: for adults with already declined IC, sarcopenia development is driven by the bidirectional vicious cycle between multi-domain functional impairment and muscle deterioration, rather than the physiological age-related muscle loss that predominates in the general population. This means that the weight and predictive value of risk factors vary significantly between the two populations, and general population models often fail to achieve sufficient sensitivity and specificity in the IC-declined subgroup, leading to unacceptably high missed diagnosis rates in this high-risk population. Therefore, a dedicated, high-sensitivity screening instrument specifically tailored to individuals with declined IC is urgently needed for targeted early intervention. Most studies focus on general middle-aged and older adult populations and thus fail to address the unique risk profile of individuals with IC decline. Consequently, this subgroup, in which sarcopenia prevalence and progression rates are substantially higher, is overlooked [13]. Third, previous prediction frameworks have not fully integrated the multidimensional nature of IC, as emphasized in the AWGS 2025 Consensus.

To address these gaps, this study will develop and validate a ML-based diagnostic model for sarcopenia, in strict accordance with the AWGS 2025 criteria, focusing on middle-aged and older adults with declined IC. Specifically, we aim to: (1) construct models using multiple ML algorithms that integrate multidimensional IC domains, demographics, and other factors; (2) identify and quantify key predictive factors, emphasizing IC-related indicators; and (3) compare algorithm performance to select the optimal tool. This work seeks to enable early risk identification, inform targeted interventions, and advance the practical implementation of the WHO ICOPE framework.

## Materials and methods

### Study design and participants

The data for this study were sourced from the China Health and Retirement Longitudinal Study (CHARLS), a nationally representative longitudinal survey targeting Chinese residents aged 45 and above. CHARLS adopts the framework of the Health and Retirement Study (HRS) and follows internationally harmonized protocols. Its sampling strategy utilized a stratified, multi-stage, probability-proportional-to-size (PPS) random sampling design. The stratification was based on county-level per-capita GDP, distinguishing between urban and rural areas. The sampling proceeded progressively through the county/district, village/community, and household levels. The baseline survey was conducted in 2011–2012, with four follow-up waves administered in 2013, 2015, 2018, and 2020. Given the CHARLS dataset’s extensive prior use, established validation, and adherence to international standards, additional reliability or validity assessments were deemed unnecessary for this analysis. To ensure national representativeness, the baseline survey encompassed 150 counties/districts and 450 villages/urban communities, resulting in a sample of 10,257 households and 17,708 individual respondents. Consequently, the dataset provides a robust representation of the demographic and socioeconomic profile of China’s middle-aged and older population.

**Fig. 1:**
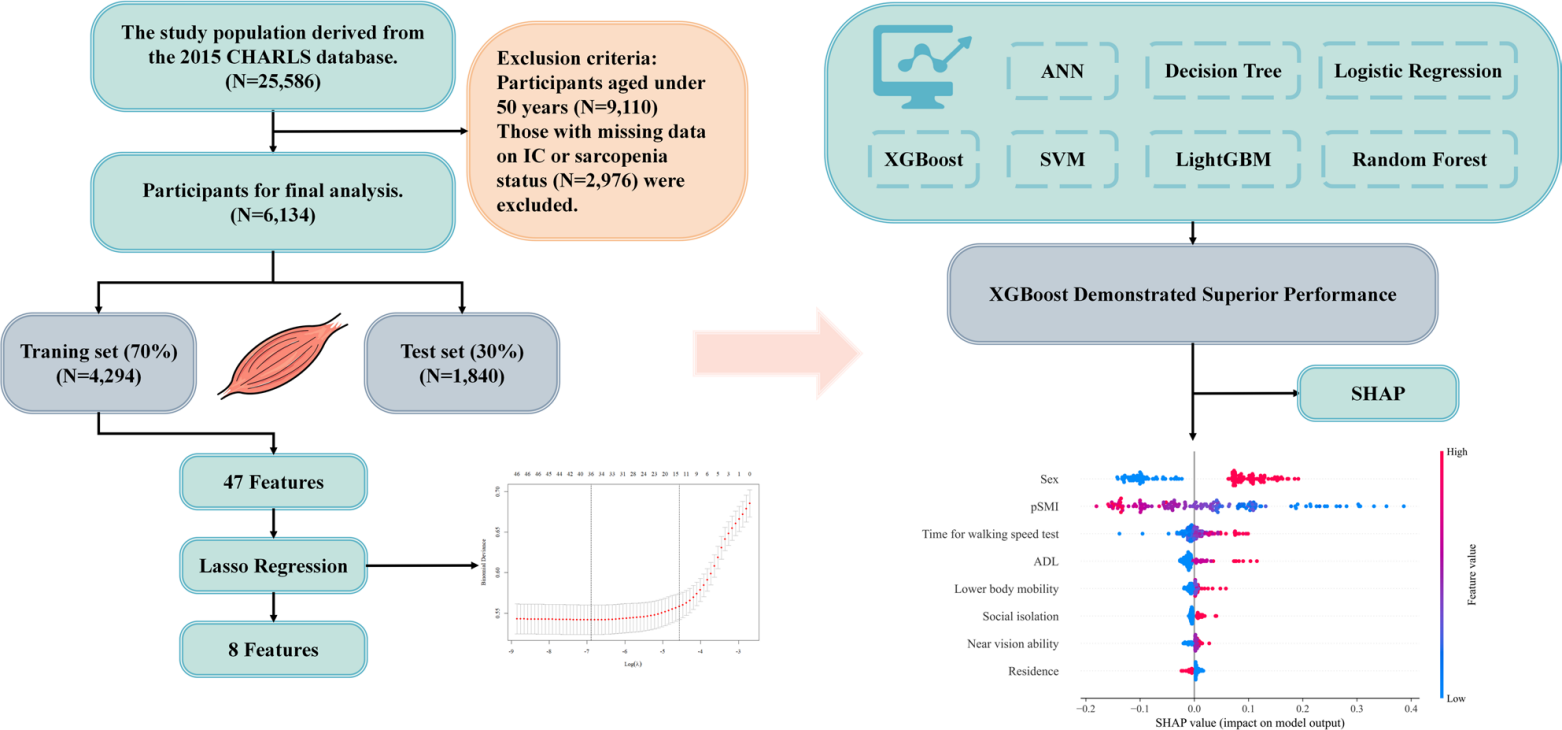
Schematic diagram of the study design. Notes: IC: Intrinsic Capacity; CHARLS: China Health and Retirement Longitudinal Study; LASSO: Least Absolute Shrinkage and Selection Operator; ANN: Artificial Neural Network; XGBoost: eXtreme Gradient Boosting; SVM: Support Vector Machine; SHAP: SHapley Additive exPlanations

This study utilized data from the third wave of the CHARLS, which was conducted in 2015. The inclusion criteria were as follows: (1) age 50 years or older; (2) presence of a decline in IC; (3) complete data available for sarcopenia assessment in accordance with the AWGS 2025 criteria. The exclusion criteria were: (1) age below 50 years; (2) missing data on IC or sarcopenia. A flowchart detailing the sample selection process and the final sample size is provided in Fig. 1.

### Outcome variables

#### Diagnosis of IC

IC was defined as a composite construct aligned with WHO framework, encompassing five core conceptual domains: vitality, cognition, locomotion, psychological function, and sensory function. Notably, the sensory domain was measured via two independent items (hearing and vision), while each of the other four domains was measured via one single item. This resulted in a total of six binary scoring items. Each domain was dichotomously coded as either preserved function (score of 1) or having an identifiable deficit (score of 0). A summary IC score (ranging from 0 to 6) was calculated by summing the domain scores, with higher values indicating better overall functional capacity. Based on prior validation studies [14, 15], a total score of ≤ 5 served as the threshold for defining declined IC.

1. Locomotion: Preserved function (score of 1) was defined as the ability to independently complete five sit-to-stand cycles within 14 s. Inability to complete the task within this time limit was scored as 0 [16].
2. Vitality: This domain was assessed via body mass index (BMI) using criteria adapted from the Malnutrition Universal Screening Tool (MUST) [12, 17]. A BMI ≥ 18.5 kg/m² indicated no nutritional risk and was scored 1, whereas a BMI < 18.5 kg/m² indicated nutritional risk and was scored 0.
3. Sensory Function: (a) Hearing: Assessed via a single self-report item (“How is your hearing?”). Responses of “fair,” “good,” “very good,” or “excellent” were scored 1; a response of “poor” was scored 0. (b) Vision: Assessed using two questions: “How well do you perceive distant objects?” and “How well do you perceive nearby objects?” A score of 1 required responses of at least “fair” to both questions. A response of “poor” to either question resulted in a score of 0.
4. Cognition: Cognitive function was assessed using the Telephone Interview for Cognitive Status (TICS) [18]. The TICS evaluates two components: a) Memory: Participants were asked to immediately recall and then again after delays of two and four minutes a list of 10 unrelated words. The maximum score for this component was 20.b) Mental Status: This component comprised three tasks: (i) Orientation (5 points): identifying the current date and season; (ii) Calculation (5 points): serially subtracting 7 from 100 five times; and (iii) Visuoconstruction (1 point): copying a drawing of two intersecting pentagons. The maximum score for mental status was 11. A domain-specific threshold for cognitive decline was defined as one standard deviation below the sample mean score. A final cognitive domain score of 1 was assigned only if performance exceeded this threshold in both memory and mental status components. A score of 0 indicated decline in one or both components.
5. Psychological Function: Depressive symptoms were assessed using the Center for Epidemiologic Studies Depression Scale (CES-D) [19]. Participants with a CES-D score < 12 were considered to have no significant depressive symptoms and received a score of 1 for this domain. A score of ≥ 12 indicated clinically relevant depressive symptoms and was coded as 0.

#### Diagnosis of sarcopenia

Sarcopenia was assessed according to the AWGS 2025 consensus. The diagnostic algorithm comprises two core components: appendicular skeletal muscle mass (ASM) and muscle strength. Sarcopenia is diagnosed by the concurrent presence of both low ASM and low muscle strength. This dual-requirement approach enhances diagnostic specificity by ensuring that muscle weakness is associated with reduced muscle mass.

Handgrip strength (kg) was measured for both the dominant and non-dominant hands using a YuejianTM WL-1000 dynamometer (Nantong Yuejian Physical Measurement Instrument Co., Ltd., China). Participants were instructed to squeeze the device as hard as possible [20]. Low muscle strength was defined according to AWGS 2025 thresholds: <28 kg for men and < 18 kg for women aged ≥ 65 years; <34 kg for men and < 20 kg for women aged 50–64 years.

ASM was estimated using a validated anthropometric equation developed for Chinese residents: ASM = 0.193 × weight (kg) + 0.107 × height (cm) − 4.157 × sex (male = 1, female = 0) − 0.037 × age (years) − 2.631 [21]. The estimated ASM showed strong agreement with measurements from dual-energy X-ray absorptiometry (DXA). Low ASM was defined using the ASM/height² index (kg/m²) according to AWGS 2025 DXA-referenced thresholds: for individuals aged ≥ 65 years, < 7.0 kg/m² for men and < 5.4 kg/m² for women; for those aged 50–64 years, < 7.2 kg/m² for men and < 5.5 kg/m² for women. BMI-adjusted thresholds (ASM/BMI) were also applied: for the ≥ 65 years group, < 0.73 for men and < 0.52 for women; for the 50–64 years group, < 0.80 for men and < 0.55 for women.

### Predictor variables

The analyzed socio-demographic characteristics included age, sex (male/female), marital status (married/single), usual residence (urban/rural), and educational level (below secondary high school, or secondary high school and above). Behavioral factors included smoking history (yes/no), drinking history (yes/no), social activities, and nighttime sleep duration. The latter was assessed by the question: “Over the past month, what was your average nightly sleep time (in hours)?” and referred specifically to actual nighttime sleep. Based on prior literature and professional expertise, we also considered the following indicators potentially associated with sarcopenia: • Health History & Status: History of chronic diseases (e.g., hypertension, diabetes, Cardiovascular Disease, stroke, cancer, chronic lung, liver, kidney, digestive diseases, arthritis, asthma), number of comorbidities, self-rated health, childhood health, depressive symptoms. • Function & Mobility: activities of daily living (ADL), instrumental activities of daily living (IADL), lower/upper limb mobility, time for walking speed test, 5-time sit-to-stand test, visual and hearing status. • Physiological & Body Measures: Systolic/diastolic blood pressure, pulmonary function, BMI, Body Roundness Index (BRI), number of pain sites, cognitive function. • Biomarkers & Indices: Non-high-density to high-density lipoprotein cholesterol ratio (NHHR), triglyceride-glucose index (TyG), predictive skeletal muscle mass index (pSMI). • Other Factors: Falls, hip fracture, denture use, social isolation. Specifically, self-rated health was categorized into three levels: ‘very good or excellent,’ ‘good,’ or ‘poor or fair.’ Pain sites included headache, shoulder pain, arm pain, wrist pain, finger pain, chest pain, abdominal pain, back pain, lumbar pain, hip pain, leg pain, knee pain, ankle pain, toe pain, or neck pain, and were classified into three groups based on the number of sites: no pain, single-site pain, and multisite pain. History of falls and hip fracture were dichotomized as yes/no. Childhood health status was classified into four levels: ‘very good or excellent,’ ‘good,’ ‘poor,’ or ‘fair.’ Near vision ability, distance vision ability, and hearing ability were each rated on a four-level scale: ‘excellent,’ ‘good,’ ‘poor,’ or ‘fair.’ Denture use and social isolation were also recorded as yes/no. The Body Roundness Index (BRI) is calculated using the following formula: $\:\mathrm{BRI=364.2-365.5}\times\sqrt{\mathrm{(1-(WC/2}\pi{\mathrm{)}}^{\mathrm{2}}\mathrm{/(0.5height}{\mathrm{)}}^{\mathrm{2}}\mathrm{)}}.$ Waist circumference (WC) and height were measured with a non-stretchable tape and a stadiometer, respectively, both to the nearest 0.1 cm. All measurements are in centimeters. The ADL scale assesses difficulties in performing basic self-care tasks, such as bathing, dressing, eating, transferring, indoor walking, and toileting [22]. In contrast, the IADL scale evaluates challenges in more complex daily activities, including using the telephone, managing finances, taking medications, shopping for groceries, and preparing meals [23]. Each item is scored dichotomously (1 = difficulty; 0 = no difficulty). Total scores range from 0 to 6 for ADL and from 0 to 5 for IADL. Cognitive function is assessed using a modified Chinese version of the Mini-Mental State Examination (MMSE). This test evaluates five domains: immediate memory, delayed memory, temporal orientation, calculation and attention, and figure drawing [24, 25]. Both immediate and delayed memory are assessed by recalling a list of ten words, with one point awarded for each correctly recalled word (each scoring 0–10 points). Temporal orientation assesses orientation to year, month, day, etc., through five questions (scoring 0–5 points). The calculation and attention test involves a serial subtraction of 7 task (scoring 0–5 points). The drawing task requires copying a figure (scoring 0–1 point). The scores from each domain are summed to yield a total cognitive score, which ranges from 0 to 31 points. A higher total score indicates better cognitive performance. Lower body mobility was assessed using four indicators of functional impairment: walking 100 m, climbing multiple flights of stairs, rising from a chair, and bending or squatting. One point was assigned for each impaired activity, with higher total scores indicating more severe lower limb dysfunction. Upper body mobility was evaluated through three indicators: extending both arms, lifting a 10-pound weight, and picking up a coin. The NHHR was calculated as the ratio of non-HDL-c to HDL-c (both in mg/dL), with non-HDL-c defined as total cholesterol (TC) minus HDL-c [26]. The TyG index was calculated using the formula: Ln [triglycerides (mg/dL) × glucose (mg/dL) / 2] [27]. The pSMI is calculated using a multivariate linear regression model with sex-specific equations. This model defines pSMI as a line. The specific sex-based equations are as follows:

For males: pSMI = 4.17 − (0.012 × age) + [1.24 × (Cr/CysC)] − (0.0513 × Hb) + (0.0598 × body weight)

For females: pSMI = 3.55 − (0.00765 × age) + [0.852 × (Cr/CysC)] − (0.0627 × Hb) + (0.0614 × body weight) [28]

### Data preprocessing

The dataset was randomly partitioned into training and testing subsets at a 7:3 ratio. The 7:3 split ratio was selected based on established machine learning guidelines for moderate-to-large sample sizes [29]. Riley and colleagues demonstrated that adequate training sample sizes are essential to minimize overfitting and ensure reliable predictor effect estimates [30]. With our total sample size of 6,134 participants (663 sarcopenia cases), the training set (*n* = 4,295) far exceeds the minimum sample size requirements proposed for binary outcome prediction models, while the 30% test set (*n* = 1,839) ensures sufficient power for robust performance evaluation. This ratio has been extensively employed in analogous investigations [31, 32]. Baseline characteristics were comprehensively compared between training and test sets to confirm successful randomization (Supplementary Table 2); no significant differences were observed in any of the eight final predictors (all *P* > 0.05). The training set was used for model development and cross-validation. This process aimed to comprehensively assess the model’s performance and stability while mitigating overfitting and underfitting. The test set, which was set aside at the outset, was reserved for the final evaluation to assess the model’s generalization ability on unseen data. This ensured no overlap between the two datasets.

To handle missing data, multiple imputation was performed using the “mice” package in R. Crucially, to prevent data leakage, the imputation model was fitted exclusively on the training set after data partitioning. The trained imputation model was then applied to the test set to generate imputed values. This approach reduces potential bias and enables the inclusion of all study participants in the subsequent analysis, and ensures that the test set remains a truly unseen and independent evaluation cohort.

Feature selection was conducted in two sequential, pre-specified steps on the training set only, to select robust predictors while avoiding overfitting. The final prediction model was developed using these selected features.

1. First-stage screening: Least Absolute Shrinkage and Selection Operator (LASSO) regression was applied to filter predictors associated with sarcopenia risk, identifying 12 candidate predictors with non-zero regression coefficients. The variance inflation factors (VIFs) for all 12 candidate variables were below 10, confirming the absence of severe multicollinearity.
2. Final refinement: These 12 candidate predictors were entered into a multivariable logistic regression model, and 8 key variables with statistical significance (*P* < 0.05) were finally identified as the fixed input features for all machine learning models in this study.

To identify sarcopenia status in middle-aged and older adults with declined IC, this study employed seven ML algorithms: Logistic Regression (LR), Decision Tree (DT), Support Vector Machine (SVM), Light Gradient Boosting Machine (LightGBM), eXtreme Gradient Boosting (XGBoost), Artificial Neural Network (ANN), and Random Forest (RF). Model optimization was performed using 5-fold cross-validation with 5 repetitions. The grid search method was applied for hyperparameter tuning to ensure model stability. The clinical value of the prediction model was comprehensively evaluated using multiple metrics: the area under the receiver operating characteristic curve (AUC), calibration curves, the Brier score, and decision curve analysis (DCA). Specifically, the AUC was used to assess the model’s discriminative power (i.e., its ability to differentiate between positive and negative cases). Calibration curves and the Brier score evaluated its calibration performance by measuring the agreement between predicted probabilities and actual outcomes. Decision curve analysis estimated the model’s net clinical benefit across various decision thresholds, offering practical guidance for clinical application.

### ML algorithms

Using the Python scikit-learn library, we constructed seven ML models based on the 8 final selected feature variables. The optimal hyperparameters for each model, determined via grid search, are as follows: Decision Tree: ccp_alpha = 0.0, max_depth = 5, max_features=None, min_samples_split = 20; Random Forest: n_estimators = 450, max_features = 3; XGBoost: learning_rate = 0.1, max_depth = 3, n_estimators = 50, subsample = 0.8; LightGBM: colsample_bytree = 0.6, learning_rate = 0.1, n_estimators = 100, num_leaves = 31, subsample = 0.6; SVM: C = 0.1, degree = 2, gamma=’scale’, kernel=’linear’; ANN: activation=’logistic’, hidden_layer_sizes=(100, 100). This grid-search-optimized parameter tuning effectively mitigated overfitting risks and significantly enhanced the predictive performance of all models on the dataset. The complete hyperparameter search spaces and final optimal values for each model are provided in Supplementary Table 3.

### SHAP interpretability analysis

Accurately interpreting ML models remains a significant challenge. The SHAP (SHapley Additive exPlanations) method addresses this by elucidating model predictions. Based on Shapley values from cooperative game theory—a concept for fairly distributing gains among participants—SHAP quantifies each feature’s contribution to a specific prediction. This feature attribution enhances the transparency of the model’s decision-making process, thereby helping to mitigate the “black box” problem.

### Statistical methods

Continuous variables are expressed as mean ± standard deviation and analyzed using the rank-sum test. Categorical variables are expressed as numbers (percentages) and compared using the chi-square (χ²) test. All statistical analyses in this study were conducted using R (version 4.5.1) and Python (version 3.10.4). A two-tailed test was employed, and a P-value of less than 0.05 was defined as statistically significant.

## Results

### Participant characteristics

The final analytical sample included 6,134 middle-aged and older adults (mean age: 62.58 ± 8.30 years; 51.08% female), with 663 individuals (10.81%) diagnosed with sarcopenia. Among the participants, 90.95% (*n* = 5,579) had less than a lower secondary education, 85.49% (*n* = 5,244) were married, and 63.76% (*n* = 3,911) resided in rural areas. Smokers accounted for 46.40% (*n* = 2,846) of the participants, and 46.28% (*n* = 2,839) reported consuming alcohol. Compared with the non-sarcopenia group, the sarcopenia group was significantly older and had a lower BMI, lower pSMI, poorer lung function, a slower walking speed, higher depressive symptom scores, and greater disability in both ADL and IADL (all *P* < 0.001). Specific comparative data are as follows: age (66.18 ± 8.81 vs. 62.15 ± 8.13 years), BMI (22.03 ± 9.97 vs. 24.17 ± 8.50 kg/m²), pSMI (6.47 ± 0.89 vs. 7.12 ± 0.93 kg/m²), lung function (254.66 ± 113.12 vs. 312.19 ± 119.00 L/min), time for walking speed test (3.79 ± 1.44 vs. 3.20 ± 1.09 s), depressive symptoms (13.70 ± 6.48 vs. 12.46 ± 5.68), ADL disability (0.67 ± 1.23 vs. 0.38 ± 0.88), and IADL disability (0.70 ± 1.14 vs. 0.40 ± 0.88). Significant between-group differences (*P* < 0.05) were observed for most variables. However, no significant differences were found for drinking status, cancer, stroke, or liver disease (*P* > 0.05). The specific baseline characteristics of the participants are presented in Table 1.

**Table 1:** Basic characteristics of study participants

Variables	Overall(*n* = 6,134)	Non-Sarcopenia(*n* = 5,471)	Sarcopenia(*n* = 663)	*P*
Sex (%)				< 0.001
Male	3001 (48.92)	2630 (48.07)	371 (55.96)	
Female	3133 (51.08)	2841 (51.93)	292 (44.04)	
Education_level (%)				< 0.001
Secondary or above	555 (9.05)	520 (9.50)	35 (5.28)	
Less than lower secondary education	5579 (90.95)	4951 (90.50)	628 (94.72)	
Marital (%)				< 0.001
Married	5244 (85.49)	4726 (86.38)	518 (78.13)	
Single	890 (14.51)	745 (13.62)	145 (21.87)	
Residence (%)				< 0.001
Village	3911 (63.76)	3424 (62.58)	487 (73.45)	
City/town	2223 (36.24)	2047 (37.42)	176 (26.55)	
Smoking (%)				0.002
Smoker	2846 (46.40)	2500 (45.70)	346 (52.19)	
Non-smoker	3288 (53.60)	2971 (54.30)	317 (47.81)	
Drinking (%)				0.146
Drinking	2839 (46.28)	2514 (45.95)	325 (49.02)	
Non-drinking	3295 (53.72)	2957 (54.05)	338 (50.98)	
Self-reported health(%)				0.222
Poor/fair	4992 (81.38)	4436 (81.08)	556 (83.86)	
Good	545 (8.88)	494 (9.03)	51 (7.69)	
Very good or excellent	597 (9.73)	541 (9.89)	56 (8.45)	
High pressure (%)				0.175
Yes	2064 (33.65)	1857 (33.94)	207 (31.22)	
No	4070 (66.35)	3614 (66.06)	456 (68.78)	
Diabetes (%)				0.004
Yes	649 (10.58)	601 (10.99)	48 (7.24)	
No	5485 (89.42)	4870 (89.01)	615 (92.76)	
Cancer (%)				0.746
Yes	115 (1.87)	101 (1.85)	14 (2.11)	
No	6019 (98.13)	5370 (98.15)	649 (97.89)	
Lung disease (%)				< 0.001
Yes	963 (15.70)	813 (14.86)	150 (22.62)	
No	5171 (84.30)	4658 (85.14)	513 (77.38)	
Cardiovascular Disease(%)				0.141
Yes	1171 (19.09)	1059 (19.36)	112 (16.89)	
No	4963 (80.91)	4412 (80.64)	551 (83.11)	
Stroke (%)				0.552
Yes	224 (3.65)	203 (3.71)	21 (3.17)	
No	5910 (96.35)	5268 (96.29)	642 (96.83)	
Arthritis (%)				0.001
Yes	2773 (45.21)	2434 (44.49)	339 (51.13)	
No	3361 (54.79)	3037 (55.51)	324 (48.87)	
Dyslipidemia (%)				0.006
yes	1174 (19.14)	1074 (19.63)	100 (15.08)	
no	4960 (80.86)	4397 (80.37)	563 (84.92)	
Liver disease (%)				0.303
Yes	444 (7.24)	403 (7.37)	41 (6.18)	
No	5690 (92.76)	5068 (92.63)	622 (93.82)	
Kidney disease (%)				0.06
Yes	694 (11.31)	604 (11.04)	90 (13.57)	
No	5440 (88.69)	4867 (88.96)	573 (86.43)	
Stomach digestive disease (%)				< 0.001
Yes	1994 (32.51)	1732 (31.66)	262 (39.52)	
No	4140 (67.49)	3739 (68.34)	401 (60.48)	
Asthma (%)				< 0.001
Yes	422 (6.88)	354 (6.47)	68 (10.26)	
No	5712 (93.12)	5117 (93.53)	595 (89.74)	
Pain location (%)				< 0.001
No pain	3962 (64.59)	3592 (65.66)	370 (55.81)	
Single-site pain	279 (4.55)	239 (4.37)	40 (6.03)	
Multiple-site pain	1893 (30.86)	1640 (29.98)	253 (38.16)	
Fall (%)				0.048
Yes	1206 (19.66)	1056 (19.30)	150 (22.62)	
No	4928 (80.34)	4415 (80.70)	513 (77.38)	
Fractured hip (%)				0.002
Yes	148 (2.41)	120 (2.19)	28 (4.22)	
No	5986 (97.59)	5351 (97.81)	635 (95.78)	
Childhood health (%)				0.022
Poor	798 (13.01)	695 (12.70)	103 (15.54)	
Fair	2410 (39.29)	2131 (38.95)	279 (42.08)	
Good	1801 (29.36)	1626 (29.72)	175 (26.40)	
Excellent	1125 (18.34)	1019 (18.63)	106 (15.99)	
Distance vision ability (%)				0.382
Poor	2008 (32.74)	1782 (32.57)	226 (34.09)	
Fair	3272 (53.34)	2924 (53.45)	348 (52.49)	
Good	741 (12.08)	659 (12.05)	82 (12.37)	
Excellent	113 (1.84)	106 (1.94)	7 (1.06)	
Near vision ability (%)				0.006
Poor	2009 (32.75)	1832 (33.49)	177 (26.70)	
Fair	3172 (51.71)	2802 (51.22)	370 (55.81)	
Good	841 (13.71)	739 (13.51)	102 (15.38)	
Excellent	112 (1.83)	98 (1.79)	14 (2.11)	
Hearing (%)				0.386
Poor	1270 (20.70)	1145 (20.93)	125 (18.85)	
Fair	3796 (61.88)	3385 (61.87)	411 (61.99)	
Good	966 (15.75)	849 (15.52)	117 (17.65)	
Excellent	102 (1.66)	92 (1.68)	10 (1.51)	
Dentures (%)				< 0.001
Yes	385 (6.28)	319 (5.83)	66 (9.95)	
No	5749 (93.72)	5152 (94.17)	597 (90.05)	
Social isolation (%)				< 0.001
Yes	1906 (31.07)	1624 (29.68)	282 (42.53)	
No	4228 (68.93)	3847 (70.32)	381 (57.47)	
Age (mean (SD))	62.58 (8.30)	62.15 (8.13)	66.18 (8.81)	< 0.001
Sleep time (mean (SD))	6.10 (1.83)	6.11 (1.82)	6.03 (1.87)	0.279
BMI (mean (SD))	23.94 (8.70)	24.17 (8.50)	22.03 (9.97)	< 0.001
BRI (mean (SD))	4.33 (2.44)	4.38 (2.39)	3.95 (2.79)	< 0.001
ADL (mean (SD))	0.41 (0.92)	0.38 (0.88)	0.67 (1.23)	< 0.001
IADL (mean (SD))	0.43 (0.92)	0.40 (0.88)	0.70 (1.14)	< 0.001
Comorbidity (mean (SD))	2.06 (1.75)	2.05 (1.76)	2.17 (1.66)	0.096
Depressive (mean (SD))	12.60 (5.78)	12.46 (5.68)	13.70 (6.48)	< 0.001
Systoblood pressure (mean (SD))	127.64 (20.25)	127.84 (20.10)	125.98 (21.41)	0.025
Diastoblood pressure (mean (SD))	74.64 (11.78)	74.91 (11.74)	72.39 (11.92)	< 0.001
Lung function (mean (SD))	305.97 (119.71)	312.19 (119.00)	254.66 (113.12)	< 0.001
Cognitive score (mean (SD))	10.79 (3.42)	10.90 (3.38)	9.82 (3.59)	< 0.001
Lower body mobility (mean (SD))	1.23 (1.21)	1.20 (1.20)	1.51 (1.30)	< 0.001
Time for walking speed test (mean (SD))	3.27 (1.15)	3.20 (1.09)	3.79 (1.44)	< 0.001
5 times sit-to-stand test (mean (SD))	10.03 (3.96)	9.92 (3.90)	10.96 (4.36)	< 0.001
Upper body mobility (mean (SD))	0.30 (0.61)	0.28 (0.59)	0.47 (0.74)	< 0.001
NHHR (mean (SD))	2.68 (0.91)	2.71 (0.92)	2.41 (0.83)	< 0.001
Tyg (mean (SD))	8.69 (0.63)	8.71 (0.63)	8.49 (0.61)	< 0.001
pSMI (mean (SD))	7.05 (0.95)	7.12 (0.93)	6.47 (0.89)	< 0.001

### Feature selection via LASSO and logistic regression

**Fig. 2:**
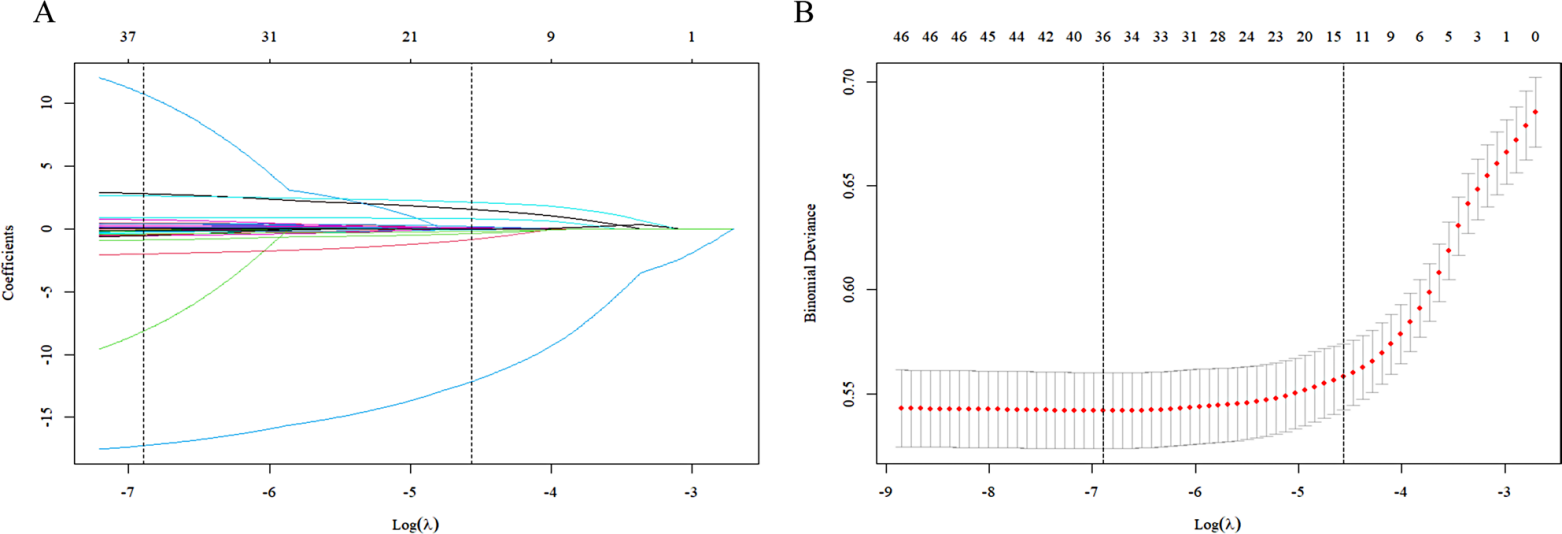
Visualization of variable selection via the LASSO regression model. **A** Coefficient profile plot. Each colored line represents the standardized regression coefficient trajectory of one predictor as log(λ) increases. The left vertical dashed line indicates λ.min (the value yielding minimum cross-validation error); the right vertical dashed line indicates λ.1SE (the largest λ within 1 standard error of the minimum, selected for parsimony). **B** Cross-validation plot for the LASSO model. The x-axis (bottom) represents log(λ) and the top axis indicates the corresponding number of non-zero coefficients. The y-axis represents binomial deviance. The left and right vertical dashed lines correspond to λ.min and λ.1SE, respectively. λ, tuning parameter; LASSO, Least Absolute Shrinkage and Selection Operator

As the regularization parameter (lambda) decreases in the LASSO path plot (Fig. 2A), the number of selected variables increases, illustrating the trade-off between model complexity and predictive accuracy. The cross-validation error for each lambda value was analyzed to identify the optimal value (Fig. 2B). Based on the lambda.1SE criterion (λ = 0.010; see Fig. 2), 12 predictive variables were selected. The variance inflation factors (VIFs) for all selected variables were below 10, confirming the absence of multicollinearity. These 12 candidate predictors identified by LASSO were then entered into a multivariable logistic regression model. The full results for all 12 variables are provided in Supplementary Table 4. Among them, eight variables remained statistically significant (*P* < 0.05) and were retained as the final predictors for machine learning model construction. The regression coefficients and odds ratios for these eight significant variables are presented in Table 2. Finally, applying a significance threshold of *P* < 0.05, eight variables were identified from the logistic regression results (Table 2): sex, residence, near vision ability, social isolation, ADL, lower body mobility, time for walking speed test, and pSMI.

**Table 2:** Results of variable selection based on binary logistic regression

Variable	OR(95%CI)	*P* value
Sex	10.722 (7.786–14.766)	< 0.01
Residence	0.780 (0.612–0.994)	0.045
Near vision ability	1.153 (1.007–1.319)	0.040
Social isolation	1.276 (1.024–1.592)	0.030
ADL	1.196 (1.051–1.360)	0.006
Lower body mobility	1.137 (1.024–1.263)	0.017
Time for walking speed test	1.232 (1.131–1.343)	< 0.01
pSMI	0.180 (0.146–0.223)	< 0.01

### Model development and comparison

**Fig. 3:**
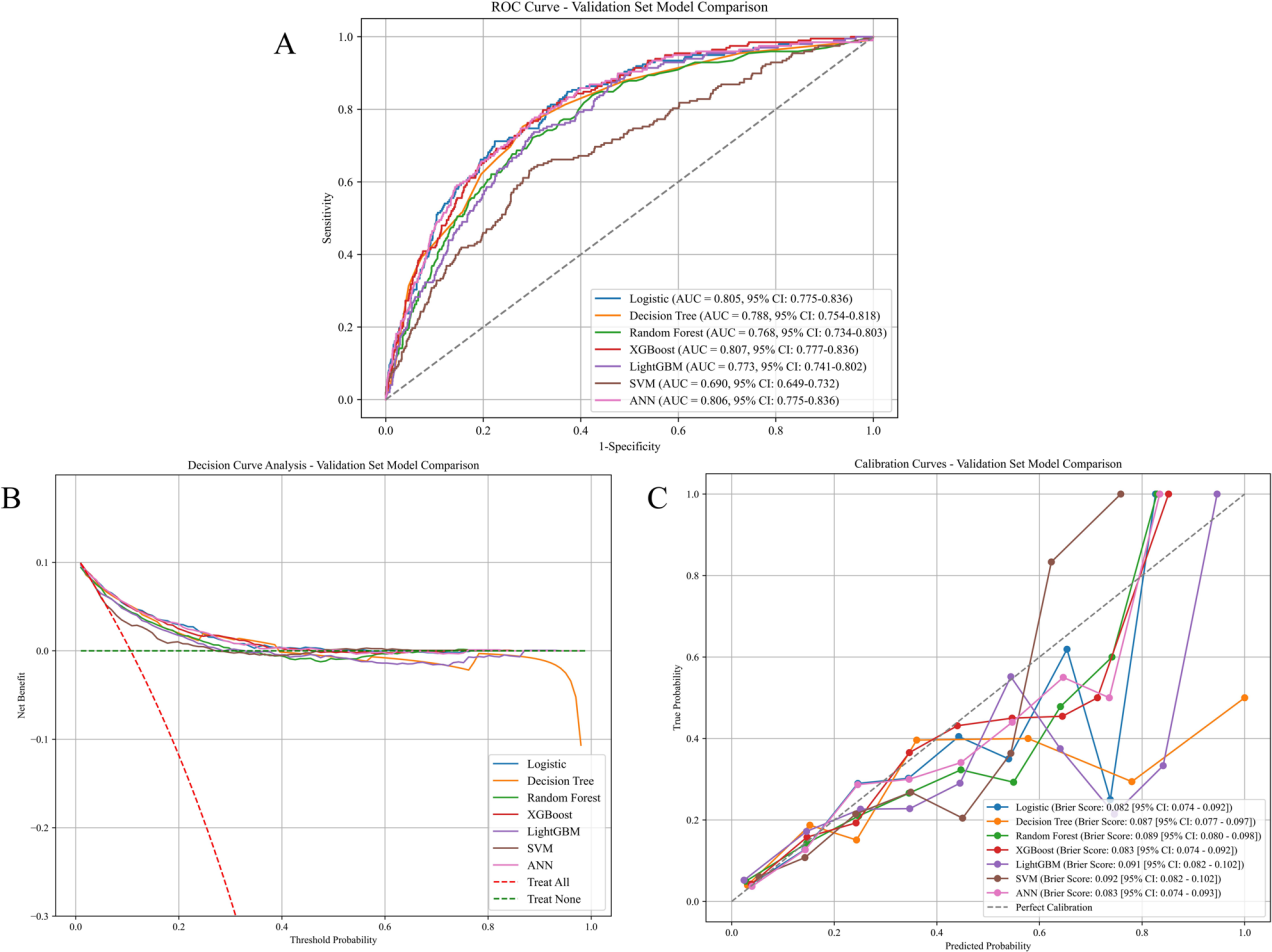
Comprehensive evaluation of ML models. **A** Receiver operating characteristic (ROC) curves. Each colored line represents one model; the diagonal dashed line represents chance-level performance (AUC = 0.5). **B** Calibration curves. Solid colored lines represent the predicted probability of sarcopenia against the observed frequency; the dashed diagonal line represents perfect calibration. **C** Decision curve analysis (DCA). Solid colored lines represent the net benefit of each model across threshold probabilities; the horizontal gray dashed line represents the ‘treat-none’ strategy (net benefit = 0), and the diagonal gray dashed line represents the ‘treat-all’ strategy. AUC: area under the curve; ROC: receiver operating characteristic; DCA: decision curve analysis

We evaluated seven ML algorithms on the testing set (Fig. 3). Model performance was assessed using three complementary metrics: (1) discriminative ability, evaluated via receiver operating characteristic (ROC) curve analysis; (2) calibration, assessed using calibration curves and the Brier score; and (3) clinical utility, determined via DCA. Given the imbalanced distribution of sarcopenia in our cohort (10.8% positive cases), we further supplemented a panel of clinically relevant performance metrics, including positive predictive value (PPV, precision), negative predictive value (NPV), F1-score, and Cohen’s Kappa (Supplementary Table 1). These metrics were used to comprehensively evaluate the model’s real-world classification performance beyond AUC, which may be misleading for imbalanced datasets, and to clarify the model’s clinical applicability in sarcopenia screening. Regarding discriminative performance, all models demonstrated moderate to excellent classification capability, with AUC values ranging from 0.690 to 0.807. XGBoost achieved the highest AUC (0.807; 95% CI: 0.777–0.836), closely followed by the artificial neural network (ANN) (0.806; 95% CI: 0.775–0.836) and logistic regression (0.805; 95% CI: 0.775–0.836). LightGBM demonstrated moderate discriminative ability (AUC = 0.773; 95% CI: 0.741–0.802). The decision tree model achieved an AUC of 0.788 (95% CI: 0.754–0.818), and random forest achieved an AUC of 0.768 (95% CI: 0.734–0.803). In contrast, support vector machine (SVM) showed the lowest discriminative ability (AUC = 0.690; 95% CI: 0.649–0.732). Calibration assessment revealed satisfactory agreement between predicted probabilities and observed outcomes for all models, with Brier scores ranging from 0.082 to 0.092. For the secondary classification metrics, all models achieved high NPVs ranging from 0.923 to 0.945, indicating an extremely low missed diagnosis rate for sarcopenia. The optimal XGBoost model achieved a sensitivity of 0.551, specificity of 0.856, PPV of 0.315, NPV of 0.940, an F1-score of 0.401, and a Cohen’s Kappa of 0.306. The logistic regression and ANN models exhibited comparable balanced performance, with F1-scores of 0.430 and 0.422, respectively, and NPVs above 0.938. Logistic regression, XGBoost, and ANN demonstrated superior calibration, each with a Brier score of 0.083. In contrast, LightGBM and SVM exhibited marginally higher scores (0.091 and 0.092, respectively), indicating slightly poorer calibration. DCA showed that all ML models provided a positive net benefit across a wide range of threshold probabilities (0.0–1.0), outperforming both the “treat-all” and “treat-none” strategies. Notably, XGBoost, ANN, and logistic regression consistently provided a higher net benefit than the other algorithms across most clinically relevant threshold ranges, indicating their robust clinical utility. Based on the integrated evaluation framework, XGBoost was selected as the optimal model. This decision was based on its: (1) superior discriminative performance (highest AUC); (2) excellent calibration (Brier score = 0.083); (3) consistently highest net benefit across threshold probabilities in DCA; and (4) favorable performance on additional metrics, including an accuracy of 0.823, sensitivity of 0.551, and specificity of 0.856. Although the ANN and logistic regression achieved comparable AUC values and calibration metrics, XGBoost demonstrated marginally better overall performance. Furthermore, the ensemble nature of XGBoost enhances its robustness against overfitting and its capacity to capture complex non-linear relationships within the data, making it particularly suitable for this clinical prediction task.

### Feature importance and variables interpretation

**Fig. 4:**
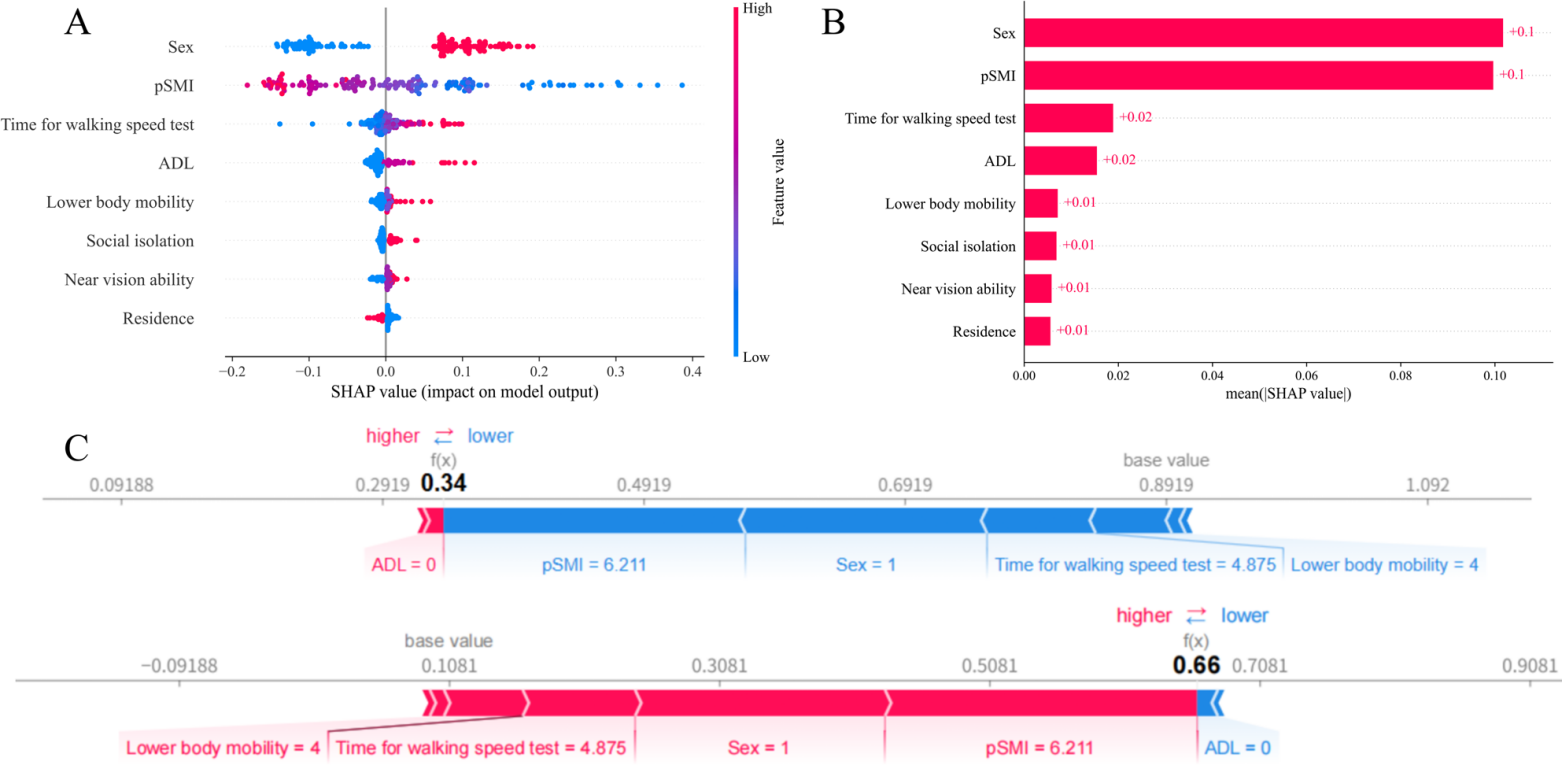
SHAP interprets the model. **A** SHAP summary plot. Each point represents one participant. The x-axis indicates the SHAP value (positive values increase the predicted probability of sarcopenia; negative values decrease it). The y-axis lists features ranked by importance (top = most important). Colors represent feature values: red indicates higher values and blue indicates lower values. **B** The mean feature importance, quantified by the magnitude of SHAP values, was calculated and ranked according to its overall contribution to predicting sarcopenia. **C** SHAP force plots for two representative cases. The base value represents the average model output over the training set. Red arrows push the prediction toward sarcopenia (higher risk); blue arrows push the prediction away from sarcopenia (lower risk). SHAP: SHapley Additive exPlanations

Figure 4A (the SHAP swarm plot) reveals the predictive mechanisms of the XGBoost model by illustrating the magnitude of each feature’s impact on sarcopenia. The X-axis represents the SHAP value. A higher SHAP value indicates a stronger positive effect of the feature on sarcopenia (i.e., an increased likelihood of sarcopenia), whereas a lower value signifies a stronger negative effect (i.e., a reduced risk of sarcopenia). The color gradient (from red to blue) represents the feature values, with red indicating higher values and blue indicating lower values. Notably, features such as Sex, pSMI, and the Time for the walking speed test have prominent impacts on model predictions, with clear directional effects on sarcopenia risk. For the categorical variable Sex (coded as 1 = male, 0 = female), higher feature values (male sex, shown in red) correlate with higher SHAP values, indicating a significantly increased risk of sarcopenia; lower feature values (female sex, shown in blue) correspond to lower SHAP values, which reduce the predicted sarcopenia risk. For example, a higher pSMI (shown in red) correlates with lower SHAP values, indicating a reduced risk of sarcopenia. Conversely, a longer Time for the walking speed test (a higher feature value) is associated with elevated SHAP values, suggesting an increased risk. Figure 4B presents the feature importance ranking for the model, as quantified by the mean absolute SHAP value. Sex and pSMI are the two most influential features, exhibiting the highest mean SHAP values, followed by the Time for the walking speed test and ADL. In contrast, Lower body mobility, Social isolation, Near vision ability, and Residence contribute minimally to the model’s predictions. To further investigate the model’s decision-making mechanism and enhance interpretability, two representative cases were selected and analyzed (Fig. 4C). The first case illustrates an individual correctly predicted as not having sarcopenia, and the second case illustrates an individual predicted as having sarcopenia.

## Discussion

This study used CHARLS data to develop, evaluate, and compare seven ML models for identifying sarcopenia status in middle-aged and older adults with declined IC. Model comparisons showed that XGBoost performed robustly across both classification and calibration metrics. The XGBoost model proved particularly suitable for this research context due to its superior classification ability, low Brier score, and well-fitted calibration curve. Furthermore, the use of SHAP values provided critical model interpretability. Thus, XGBoost can effectively identify sarcopenia risk in this population, facilitating early intervention and potentially improving health outcomes.

LASSO regression analysis identified eight key characteristic variables. As shown in Fig. 4, both a higher pSMI and urban residence were associated with a decreased risk of sarcopenia. Conversely, the following factors were significantly associated with an increased probability of sarcopenia: male sex, higher scores for lower limb mobility impairment and ADL limitations, social isolation, impaired near vision, and slow gait speed. These associations are consistent with prior research findings. Notably, the odds ratio for male sex in the present study (OR = 10.722, 95% CI: 7.786–14.766) was substantially higher than estimates reported in general older adult populations. Several factors may account for this pronounced association. First, the study cohort was restricted to individuals with already declined intrinsic capacity—a high-risk subgroup in which the pathophysiological vulnerability of males to muscle deterioration may be disproportionately amplified. In this specific population, male sex acts as an effect modifier that synergistically interacts with locomotor domain deficits (the core component of declined IC) to accelerate sarcopenia development. Second, the strict age- and sex-specific diagnostic thresholds for sarcopenia from the AWGS 2025 consensus (e.g., a handgrip strength cutoff of 34 kg for men aged 50–64 years vs. 20 kg for women in the same age group) further amplified the sex difference in sarcopenia diagnosis in our middle-aged and older cohort. Third, the multivariable model simultaneously adjusted for pSMI, an anthropometric index that partially captures muscle mass and is highly correlated with sex. By accounting for baseline muscle quantity, the independent effect of male sex on sarcopenia risk was isolated, potentially magnifying the residual sex-specific risk attributable to unmeasured factors such as hormonal status, inflammatory profiles, or neuromuscular integrity. Importantly, we carefully examined the model for potential statistical artifacts: variance inflation factors for all covariates were below 10, confirming the absence of severe multicollinearity; sensitivity analyses via stepwise regression and 5-fold cross-validation (consistent with our model construction process) yielded highly consistent effect estimates; and no coding errors were identified. Therefore, while the magnitude of the OR should be interpreted with caution and validated in external cohorts, it likely reflects a genuine, context-specific risk profile within this unique population of middle-aged and older adults with declined IC, rather than a methodological flaw. Male sex is a well-established risk factor for both the onset and progression of sarcopenia [33], a finding consistent across multiple studies. A long-term cohort study reported that male sex was significantly associated with a higher risk of sarcopenia progression (HR = 1.84) [34]. In aged C57BL/6J mice, males exhibited more pronounced age-related declines in muscle strength and physical performance, along with a higher prevalence and severity of sarcopenia, compared to age-matched females [35]. This cross-species consistency underscores sex as a fundamental biological variable in sarcopenia pathophysiology, likely mediated by sexual dimorphism in hormone levels, metabolism, and molecular signaling pathways. Furthermore, during sarcopenia progression, male mice exhibited marked reductions in mitochondrial biogenesis (e.g., lower PGC-1α expression), decreased oxidative phosphorylation complex levels, downregulation of autophagy markers (p62 and LC3), and attenuated AMPK signaling. These pathways, which are crucial for energy metabolism and cellular quality control, were more severely impaired in males. Consequently, males may be more vulnerable to age-related losses in muscle mass and function. In contrast, female mice maintained relatively normal activity in these pathways and did not show significant upregulation of atrophy-related genes (Atrogin-1 and MuRF1). These molecular-level differences may partly explain the higher prevalence and progression rate of sarcopenia in males, a pattern observed in both clinical and animal studies [35]. Consistent with other cohort studies, pSMI shows a strong inverse association with sarcopenia among middle-aged and older adults in China [28, 36]. Research indicates that each standard-deviation decrease in pSMI is associated with a 2.93-fold increase in the 4-year risk of sarcopenia. Furthermore, among adults aged ≥ 60 years, sarcopenia prevalence decreased dramatically across pSMI quartiles, from 55.3% in men and 66.6% in women in the lowest quartile to only 0.4% in the highest. These findings support pSMI as a reliable and scalable biomarker for sarcopenia. pSMI outperforms traditional indices such as the creatinine-to-cystatin C ratio (CCR) and the sarcopenia index (SI), due to its stronger correlation with muscle mass (*r* = 0.87) and grip strength (*r* = 0.59), and its ability to predict long-term mortality independent of baseline sarcopenia status. The strong association between pSMI and sarcopenia stems from its integration of key physiological domains: muscle metabolism, renal function, and systemic physiology. Serum creatinine (Cr) is a non-enzymatic breakdown product of phosphocreatine in skeletal muscle. Although Cr levels partially reflect overall muscle mass, they are strongly influenced by sex, race, and renal function. Cystatin C (CysC) is a low-molecular-weight protein produced by all nucleated cells. It is freely filtered by the glomerulus and almost completely reabsorbed and degraded by the proximal tubules. Unlike Cr, its production rate is relatively constant and less influenced by muscle mass or sex. Due to their complementary origins, metabolism, and responses to renal function and muscle mass, composite indices based on Cr and CysC can integrate information reflecting the balance between muscle metabolism and renal clearance. Therefore, such composite metrics show promise as biomarkers for assessing muscle status [37]. The inclusion of hemoglobin underscores the role of oxygen delivery in muscle homeostasis, while adjustments for body weight and age account for physiological changes associated with aging [38]. Collectively, these elements enable pSMI to capture both muscle quantity and functional integrity, underpinning its value as a biomarker for both the early diagnosis and prognosis of sarcopenia. Gait speed is a key functional marker of muscular health and overall mobility. A study of 95 frail older adults (44 with sarcopenia) analyzed eight gait parameters and found that gait speed and turn time under dual-task conditions were effective predictors of sarcopenia in this population. The combination of these two parameters produced an AUC of 0.763, with a sensitivity of 75.0% and a specificity of 74.5% [39]. These findings indicate that gait speed could be a practical and sensitive digital biomarker for the early screening of sarcopenia in older adults. A meta-analysis of seven studies demonstrated that limitations in ADL significantly increase the risk of sarcopenia in community-dwelling older adults (pooled OR = 1.49, 95% CI: 1.15–1.92, *p* < 0.001) [40]. Mechanistically, ADL limitations are linked to several underlying factors, including chronic inflammation, metabolic dysregulation, and malnutrition. Furthermore, by reducing overall physical capacity and diminishing the frequency and intensity of muscle engagement, ADL limitations can directly promote muscle loss and the progression of sarcopenia [41]. In this study, lower limb dysfunction demonstrated significant and robust predictive capability for sarcopenia onset. In contrast, indicators of upper limb dysfunction were excluded from the final model due to a lack of independent predictive value. This finding does not negate the association between upper limb function and sarcopenia. Instead, it suggests that for predicting sarcopenia onset, lower limb functional decline may be an earlier, more sensitive, and more specific clinical indicator. This observation is physiologically plausible. The lower limb muscle groups, particularly the gastrocnemius and quadriceps, are the body’s largest and most metabolically active. They are crucial for anti-gravity and load-bearing functions. Consequently, in the progression of sarcopenia, these major muscle groups are often among the first to be affected [42]. Their functional decline can thus be viewed as both an “amplifier” of disability and an objective marker that the depletion of systemic muscle reserves has reached a clinically significant threshold. Accumulating evidence indicates a significant association between social isolation and sarcopenia in older adults. A systematic review and meta-analysis of 27,437 individuals demonstrated that social isolation independently increased sarcopenia risk, with a pooled odds ratio of 1.70 (95% CI: 1.51–1.92) [43]. A longitudinal study corroborated these findings, further revealing that social isolation significantly predicted new-onset sarcopenia incidence among community-dwelling older adults (HR = 1.115, 95% CI: 1.013–1.228) [44]. Current research consistently identifies social isolation as an important and modifiable risk factor for sarcopenia in older adults, potentially mediated through psychological and cognitive pathways. Visual impairment is a recognized risk factor for sarcopenia in older adults. Substantial evidence supports a significant association between the two conditions. For example, a cross-sectional study of 403 urban older adults in Cameroon found that those with self-reported visual impairment had a significantly higher risk of sarcopenia (OR = 2.66, 95% CI: 1.29–5.48). This association remained significant after adjusting for multiple confounders and persisted even after visual correction (adjusted OR = 2.47, 95% CI: 1.18–5.17) [45]. These findings align with a systematic review of 13 observational studies, which concluded that visual impairment independently increases sarcopenia risk and is closely associated with its core components, such as low muscle mass and reduced gait speed [46]. Notably, near-vision impairment may be particularly important in this association. An analysis of US National Health and Nutrition Examination Survey (NHANES) data (*n* = 2,705) showed that both objectively measured and self-reported near-vision impairment were significantly associated with a higher risk of frailty. Frailty and sarcopenia share considerable overlap in their underlying multisystem pathological mechanisms, particularly regarding declines in muscle function [47]. The prevalence of sarcopenia shows significant urban-rural differences in the existing literature. A systematic review and meta-analysis of 66 studies (433,091 participants) found a global pooled sarcopenia prevalence of 18%, with significantly higher rates in rural (20%) than in urban populations (16%) [48]. This trend is consistently observed in regional studies from The Gambia and South Korea [49, 50]. However, in the present study, residence (urban vs. rural) emerged as a relatively weak independent predictor in the final XGBoost model, as reflected by its low mean SHAP value (Fig. 4B). This apparent discrepancy may be explained by the fact that our model simultaneously adjusted for a comprehensive set of factors that are often unevenly distributed between urban and rural settings—such as pSMI, lower body mobility, gait speed, and ADL limitations—which may capture much of the variance previously attributed to residence alone. In other words, the effect of urban-rural residence on sarcopenia risk may be largely mediated by, or confounded by, these more proximal determinants of muscle health. Once these factors are accounted for, the independent contribution of residence per se diminishes. This finding underscores the value of machine learning models in disentangling the complex interplay between socioeconomic context and individual-level physiological risk factors.

Prior research has shown that individuals with declined IC face a higher risk of sarcopenia. However, most existing studies have examined only specific dimensions—such as cognition, sensory function, vitality, psychological state, and locomotion—rather than conducting a systematic analysis of all dimensions and their interactions. Moreover, due to inconsistencies in the definition and assessment of sarcopenia across studies, direct comparison and integration of findings are challenging, which limits the generalizability of the conclusions. This study has several key strengths: (1) The data come from CHARLS, a large-scale national public database that covers multiple provinces across China. CHARLS includes a well-designed questionnaire system and rich demographic variables, ensuring strong national representativeness. (2) We used seven ML algorithms to develop a sarcopenia risk prediction model for middle-aged and older Chinese adults with reduced IC. These methods can automatically identify key predictors and capture complex nonlinear relationships, often yielding higher predictive accuracy than traditional statistical models. (3) Applying the updated diagnostic thresholds from the latest AWGS 2025 criteria, this study is one of the first to include adults aged 50–64 years, aligning with the consensus’s age range expansion for sarcopenia screening. This approach allows a more comprehensive understanding of muscle health trajectories across the lifespan and supports earlier identification and intervention.

However, this study has several limitations. First, the model relied exclusively on retrospective cross-sectional data from the single CHARLS database. While a high-quality, nationally representative database was used and the model performed well in internal validation, the cross-sectional design precludes causal inference between the identified predictors and sarcopenia, cannot determine the directional relationship between variables, and the lack of prospective follow-up data hinders the dynamic assessment and longitudinal analysis of sarcopenia progression. Meanwhile, the potential for selection bias persists despite strict inclusion and exclusion criteria. Second, the diagnosis of sarcopenia in this study relies on ASM estimated via a validated anthropometric equation for Chinese populations, rather than the gold-standard DXA measurement. Although the estimated ASM used in this study has been demonstrated to have strong agreement with DXA-measured values in prior Chinese population-based studies, the estimated values cannot fully replace the gold-standard in vivo measurement of muscle mass by DXA. Critically, we applied the AWGS 2025 DXA-referenced diagnostic thresholds to the estimated ASM values for sarcopenia case definition, which inevitably carries a potential risk of misclassification bias. This bias may further affect the calibration performance and discriminative ability of the prediction model to a certain extent. Third, the model was constructed based on 2015 CHARLS data and developed in strict accordance with the AWGS 2025 consensus criteria released a decade later. Changes in population demographic characteristics, lifestyle patterns, healthcare access and medical practice standards over time, as well as potential future updates to sarcopenia diagnostic criteria, may lead to calibration drift of the model, resulting in a decline in model performance when applied to future populations. Fourth, all predictors included in the model were derived from existing variables in the CHARLS database, and other potentially relevant biological indicators (such as muscle-specific biomarkers) and imaging parameters were omitted. Consequently, the comprehensiveness and predictive accuracy of the model may be further optimized by integrating these potential predictors.

## Conclusion

In summary, leveraging the nationally representative CHARLS cross-sectional database, this study developed and compared seven ML models to identify sarcopenia status and screen key associated factors in middle-aged and older adults experiencing IC decline. The results indicated that the XGBoost model emerged as the top-performing model, demonstrating superior predictive accuracy and stability. This study provides healthcare practitioners with an efficient and accurate tool for sarcopenia risk screening, supporting early intervention and personalized management strategies. Future research should prioritize the following directions: (1) Conduct external validation of the model in multi-center, prospective cohorts with sarcopenia diagnosed via the gold-standard DXA measurement, to verify the generalizability and robustness of the model across different populations and clinical settings; (2) Perform regular recalibration of the model based on updated population data and the latest diagnostic criteria, to mitigate the impact of potential calibration drift and maintain the long-term clinical applicability of the model; (3) Further optimize the model by integrating novel biomarkers and multi-dimensional indicators, and explore the clinical implementation pathways of the model in community-based elderly health management, to promote the early prevention and control of sarcopenia.

## Supplementary Information

Below is the link to the electronic supplementary material.

Supplementary Material 1

## Data Availability

The datasets used and analyzed in this study are publicly available from the CHARLS repository at: http://charls.pku.edu.cn.
