# Supplementary material for: Leveraging interpretable machine learning to identify sarcopenia in middle-aged and older adults with intrinsic capacity decline: an analysis of CHARLS data under AWGS 2025
Source: BMC Med Inform Decis Mak. 2026 Jun 3;26:300. doi: 10.1186/s12911-026-03606-x (PMC13450456; doi:10.1186/s12911-026-03606-x)
Supplement: Supplementary file 1 [file 12911_2026_3606_MOESM1_ESM.docx]

**Supplementary Fig.1** Scatter plot of SHAP values for each key feature. Scatter plot (SHAP dependence plot) of SHAP values for each key feature. Each panel corresponds to one of the eight final predictors. The y-axis represents the SHAP value (positive = increased sarcopenia probability; negative = decreased probability). The x-axis represents the feature value (continuous variables) or category (binary/categorical variables). The red solid line indicates the LOWESS trend of the relationship between feature value and SHAP value, and the orange dashed line at y=0 is the baseline of zero effect. The color gradient from purple/blue to yellow indicates the value of the primary feature itself, from low to high, respectively.

**Supplementary Table 1** Model performance evaluation

| Model | AUC(95%CI) | Accuracy | Sensitivity | Specificity | Youden's J index | F1-Score | PPV | NPV | Kappa |
| --- | --- | --- | --- | --- | --- | --- | --- | --- | --- |
| Logistic | 0.805 (0.775-0.836) | 0.854 | 0.510 | 0.896 | 0.406 | 0.430 | 0.371 | 0.938 | 0.349 |
| Decision Tree | 0.788 (0.754-0.818) | 0.868 | 0.394 | 0.926 | 0.320 | 0.392 | 0.390 | 0.927 | 0.318 |
| Random Forest | 0.768 (0.734-0.803) | 0.816 | 0.505 | 0.853 | 0.358 | 0.371 | 0.293 | 0.935 | 0.272 |
| XGBoost | 0.807 (0.777-0.836) | 0.823 | 0.551 | 0.856 | 0.406 | 0.401 | 0.315 | 0.940 | 0.306 |
| LightGBM | 0.773 (0.741-0.802) | 0.754 | 0.631 | 0.769 | 0.400 | 0.356 | 0.248 | 0.945 | 0.238 |
| SVM | 0.690 (0.649-0.732) | 0.799 | 0.414 | 0.846 | 0.260 | 0.308 | 0.245 | 0.923 | 0.199 |
| ANN | 0.806 (0.775-0.836) | 0.830 | 0.576 | 0.861 | 0.437 | 0.422 | 0.333 | 0.944 | 0.331 |

Note: All performance metrics were calculated based on the optimal cut-off threshold corresponding to the maximum Youden’s index of the ROC curve on the independent test set. PPV: Positive Predictive Value; NPV: Negative Predictive Value.

**Supplementary Table 2** Comparison of baseline characteristics between training and testing sets

| Variables | Overall(n=6,134) | Training set(n=4,295) | Testing set(n=1,839) | P |
| --- | --- | --- | --- | --- |
| Sex (%) |  |  |  | 0.729 |
| male | 3001 (48.92) | 2108 (49.08) | 893 (48.56) |  |
| female | 3133 (51.08) | 2187 (50.92) | 946 (51.44) |  |
| Education_level (%) |  |  |  | 0.005 |
| secondary or above | 555 ( 9.05) | 359 ( 8.36) | 196 (10.66) |  |
| less than lower secondary education | 5579 (90.95) | 3936 (91.64) | 1643 (89.34) |  |
| Marital (%) |  |  |  | 0.045 |
| married | 5244 (85.49) | 3646 (84.89) | 1598 (86.90) |  |
| single | 890 (14.51) | 649 (15.11) | 241 (13.10) |  |
| Residence (%) |  |  |  | 0.563 |
| village | 3911 (63.76) | 2728 (63.52) | 1183 (64.33) |  |
| city/town | 2223 (36.24) | 1567 (36.48) | 656 (35.67) |  |
| Smoking (%) |  |  |  | 0.586 |
| smoker | 2846 (46.40) | 2003 (46.64) | 843 (45.84) |  |
| non-smoker | 3288 (53.60) | 2292 (53.36) | 996 (54.16) |  |
| Drinking (%) |  |  |  | 0.839 |
| drinking | 2839 (46.28) | 1992 (46.38) | 847 (46.06) |  |
| non-drinking | 3295 (53.72) | 2303 (53.62) | 992 (53.94) |  |
| Self-reported health(%) |  |  |  | 0.859 |
| poor/fair | 4992 (81.38) | 3500 (81.49) | 1492 (81.13) |  |
| good | 545 ( 8.88) | 376 ( 8.75) | 169 ( 9.19) |  |
| very good or excellent | 597 ( 9.73) | 419 ( 9.76) | 178 ( 9.68) |  |
| High pressure (%) |  |  |  | 0.490 |
| yes | 2064 (33.65) | 1433 (33.36) | 631 (34.31) |  |
| no | 4070 (66.35) | 2862 (66.64) | 1208 (65.69) |  |
| Diabetes (%) |  |  |  | 0.149 |
| yes | 649 (10.58) | 438 (10.20) | 211 (11.47) |  |
| no | 5485 (89.42) | 3857 (89.80) | 1628 (88.53) |  |
| Cancer (%) |  |  |  | 0.996 |
| yes | 115 ( 1.87) | 80 ( 1.86) | 35 ( 1.90) |  |
| no | 6019 (98.13) | 4215 (98.14) | 1804 (98.10) |  |
| Lung disease (%) |  |  |  | 0.327 |
| yes | 963 (15.70) | 661 (15.39) | 302 (16.42) |  |
| no | 5171 (84.30) | 3634 (84.61) | 1537 (83.58) |  |
| Cardiovascular Disease(%) |  |  |  | 0.641 |
| yes | 1171 (19.09) | 827 (19.25) | 344 (18.71) |  |
| no | 4963 (80.91) | 3468 (80.75) | 1495 (81.29) |  |
| Stroke (%) |  |  |  | 0.587 |
| yes | 224 ( 3.65) | 161 ( 3.75) | 63 ( 3.43) |  |
| no | 5910 (96.35) | 4134 (96.25) | 1776 (96.57) |  |
| Arthritis (%) |  |  |  | 0.905 |
| yes | 2773 (45.21) | 1939 (45.15) | 834 (45.35) |  |
| no | 3361 (54.79) | 2356 (54.85) | 1005 (54.65) |  |
| Dyslipidemia (%) |  |  |  | 0.146 |
| yes | 1174 (19.14) | 801 (18.65) | 373 (20.28) |  |
| no | 4960 (80.86) | 3494 (81.35) | 1466 (79.72) |  |
| Liver disease (%) |  |  |  | 0.175 |
| yes | 444 ( 7.24) | 324 ( 7.54) | 120 ( 6.53) |  |
| no | 5690 (92.76) | 3971 (92.46) | 1719 (93.47) |  |
| Kidney disease (%) |  |  |  | 0.237 |
| yes | 694 (11.31) | 472 (10.99) | 222 (12.07) |  |
| no | 5440 (88.69) | 3823 (89.01) | 1617 (87.93) |  |
| Stomach digestive disease (%) |  |  |  | 0.276 |
| yes | 1994 (32.51) | 1415 (32.95) | 579 (31.48) |  |
| no | 4140 (67.49) | 2880 (67.05) | 1260 (68.52) |  |
| Asthma (%) |  |  |  | 0.153 |
| yes | 422 ( 6.88) | 282 ( 6.57) | 140 ( 7.61) |  |
| no | 5712 (93.12) | 4013 (93.43) | 1699 (92.39) |  |
| Pain location (%) |  |  |  | 0.701 |
| no pain | 3962 (64.59) | 2760 (64.26) | 1202 (65.36) |  |
| single-site pain | 279 ( 4.55) | 196 ( 4.56) | 83 ( 4.51) |  |
| multiple-site pain | 1893 (30.86) | 1339 (31.18) | 554 (30.13) |  |
| Fall (%) |  |  |  | 0.159 |
| yes | 1206 (19.66) | 865 (20.14) | 341 (18.54) |  |
| no | 4928 (80.34) | 3430 (79.86) | 1498 (81.46) |  |
| Fractured hip (%) |  |  |  | 0.482 |
| yes | 148 ( 2.41) | 108 ( 2.51) | 40 ( 2.18) |  |
| no | 5986 (97.59) | 4187 (97.49) | 1799 (97.82) |  |
| Childhood health (%) |  |  |  | 0.854 |
| poor | 798 (13.01) | 561 (13.06) | 237 (12.89) |  |
| fair | 2410 (39.29) | 1688 (39.30) | 722 (39.26) |  |
| good | 1801 (29.36) | 1249 (29.08) | 552 (30.02) |  |
| excellent | 1125 (18.34) | 797 (18.56) | 328 (17.84) |  |
| Distance vision ability (%) |  |  |  | 0.776 |
| poor | 2008 (32.74) | 1391 (32.39) | 617 (33.55) |  |
| fair | 3272 (53.34) | 2307 (53.71) | 965 (52.47) |  |
| good | 741 (12.08) | 516 (12.01) | 225 (12.23) |  |
| excellent | 113 ( 1.84) | 81 ( 1.89) | 32 ( 1.74) |  |
| Near vision ability (%) |  |  |  | 0.056 |
| poor | 2009 (32.75) | 1454 (33.85) | 555 (30.18) |  |
| fair | 3172 (51.71) | 2189 (50.97) | 983 (53.45) |  |
| good | 841 (13.71) | 575 (13.39) | 266 (14.46) |  |
| excellent | 112 ( 1.83) | 77 ( 1.79) | 35 ( 1.90) |  |
| Hearing (%) |  |  |  | 0.646 |
| poor | 1270 (20.70) | 897 (20.88) | 373 (20.28) |  |
| fair | 3796 (61.88) | 2653 (61.77) | 1143 (62.15) |  |
| good | 966 (15.75) | 679 (15.81) | 287 (15.61) |  |
| excellent | 102 ( 1.66) | 66 ( 1.54) | 36 ( 1.96) |  |
| Dentures (%) |  |  |  | 0.11 |
| yes | 385 ( 6.28) | 284 ( 6.61) | 101 ( 5.49) |  |
| no | 5749 (93.72) | 4011 (93.39) | 1738 (94.51) |  |
| Social isolation (%) |  |  |  | 0.308 |
| yes | 1906 (31.07) | 1352 (31.48) | 554 (30.13) |  |
| no | 4228 (68.93) | 2943 (68.52) | 1285 (69.87) |  |
| Age (mean (SD)) | 62.58 (8.30) | 62.63 (8.33) | 62.48 (8.24) | 0.509 |
| Sleep time (mean (SD)) | 6.10 (1.83) | 6.10 (1.83) | 6.09 (1.82) | 0.836 |
| BMI (mean (SD)) | 23.94 (8.70) | 23.93 (8.94) | 23.94 (8.12) | 0.965 |
| BRI (mean (SD)) | 4.33 (2.44) | 4.34 (2.43) | 4.32 (2.46) | 0.726 |
| ADL (mean (SD)) | 0.41 (0.92) | 0.41 (0.91) | 0.43 (0.97) | 0.431 |
| IADL (mean (SD)) | 0.43 (0.92) | 0.43 (0.92) | 0.44 (0.93) | 0.884 |
| Comorbidity (mean (SD)) | 2.06 (1.75) | 2.05 (1.74) | 2.09 (1.76) | 0.417 |
| Depressive (mean (SD)) | 12.60 (5.78) | 12.73 (5.86) | 12.29 (5.57) | 0.007 |
| Systoblood pressure (mean (SD)) | 127.64 (20.25) | 127.46 (20.30) | 128.05 (20.14) | 0.301 |
| Diastoblood pressure (mean (SD)) | 74.64 (11.78) | 74.48 (11.83) | 75.01 (11.66) | 0.104 |
| Lung function (mean (SD)) | 305.97 (119.71) | 306.18 (120.37) | 305.48 (118.20) | 0.835 |
| Cognitive score (mean (SD)) | 10.79 (3.42) | 10.76 (3.43) | 10.85 (3.38) | 0.379 |
| Lower body mobility (mean (SD)) | 1.23 (1.21) | 1.24 (1.21) | 1.20 (1.23) | 0.229 |
| Time for walking speed test (mean (SD)) | 3.27 (1.15) | 3.28 (1.16) | 3.24 (1.13) | 0.207 |
| 5 times sit-to-stand test (mean (SD)) | 10.03 (3.96) | 10.06 (3.92) | 9.98 (4.07) | 0.478 |
| Upper body mobility (mean (SD)) | 0.30 (0.61) | 0.29 (0.60) | 0.30 (0.63) | 0.544 |
| NHHR (mean (SD)) | 2.68 (0.91) | 2.70 (0.93) | 2.64 (0.85) | 0.032 |
| Tyg (mean (SD)) | 8.69 (0.63) | 8.69 (0.63) | 8.67 (0.63) | 0.245 |
| pSMI (mean (SD)) | 7.05 (0.95) | 7.05 (0.95) | 7.04 (0.95) | 0.698 |

**Supplementary Table 3** Hyperparameter search spaces and optimal values for each machine learning model.

| Model | Hyperparameter | Search Space | Optimal Value |
| --- | --- | --- | --- |
| Decision Tree | max_depth | {3, 5, 10, None} | 5 |
|  | min_samples_split | {5, 10, 20} | 20 |
|  | max_features | {'sqrt', None} | None |
|  | ccp_alpha | {0.0, 0.01, 0.1} | 0 |
| Random Forest | n_estimators | {50, 100, 150, 200, 250, 300, 350, 400, 450} | 450 |
|  | max_features | {2, 3} | 3 |
| XGBoost | learning_rate | {0.01, 0.1, 0.2} | 0.1 |
|  | max_depth | {3, 5, 10} | 3 |
|  | n_estimators | {50, 100, 200} | 50 |
|  | subsample | {0.6, 0.8, 1.0} | 0.8 |
| LightGBM | learning_rate | {0.1, 0.2} | 0.1 |
|  | num_leaves | {31, 50, 100} | 31 |
|  | n_estimators | {100} | 100 |
|  | subsample | {0.6, 0.8, 1.0} | 0.6 |
|  | colsample_bytree | {0.6, 0.8, 1.0} | 0.6 |
| SVM | C | {0.1} | 0.1 |
|  | kernel | {'linear', 'rbf'} | 'linear' |
|  | gamma | {'scale', 'auto', 0.01} | 'scale' |
|  | degree | {2, 3, 4} | 2 |
| ANN | hidden_layer_sizes | {(25,), (50,), (100,), (10, 10), (50, 50), (100, 100), (50, 50, 50)} | (100, 100) |
|  | activation | {'relu', 'tanh', 'logistic'} | 'logistic' |

**Supplementary Table 4** Full results of multivariable logistic regression for all 12 candidate predictors identified by LASSO.

| Variable | Beta | SE | Wald | OR | Lower CI | Upper CI | P value |
| --- | --- | --- | --- | --- | --- | --- | --- |
| Sex | 2.372 | 0.163 | 14.532 | 10.722 | 7.786 | 14.766 | <0.01 |
| Residence | -0.248 | 0.124 | -2.008 | 0.780 | 0.612 | 0.994 | 0.045 |
| Near vision ability | 0.142 | 0.069 | 2.057 | 1.153 | 1.007 | 1.319 | 0.040 |
| Social isolation | 0.244 | 0.113 | 2.167 | 1.276 | 1.024 | 1.592 | 0.030 |
| ADL | 0.179 | 0.066 | 2.725 | 1.196 | 1.051 | 1.360 | 0.006 |
| IADL | 0.035 | 0.064 | 0.547 | 1.036 | 0.913 | 1.175 | 0.584 |
| Lung function | -0.001 | 0.001 | -1.498 | 0.999 | 0.998 | 1.000 | 0.134 |
| Lower body mobility | 0.128 | 0.054 | 2.394 | 1.137 | 1.024 | 1.263 | 0.017 |
| Time for walking speed test | 0.209 | 0.044 | 4.767 | 1.232 | 1.131 | 1.343 | <0.01 |
| NHHR | -0.105 | 0.076 | -1.377 | 0.900 | 0.775 | 1.046 | 0.169 |
| TyG | -0.068 | 0.114 | -0.601 | 0.934 | 0.747 | 1.167 | 0.548 |
| pSMI | -1.712 | 0.109 | -15.775 | 0.180 | 0.146 | 0.223 | <0.01 |
